# Crystal structure of poly[bis­(ammonium) [bis­(μ_4_-benzene-1,3,5-tri­carboxyl­ato)dizincate] 1-methyl­pyrrolidin-2-one disolvate]

**DOI:** 10.1107/S2056989016007027

**Published:** 2016-04-29

**Authors:** Carlos Ordonez, Marina S. Fonari, Qiang Wei, Tatiana V. Timofeeva

**Affiliations:** a Department of Biology & Chemistry, New Mexico Highlands University, Las Vegas, NM 87701, USA; b Institute of Applied Physics, Academy of Sciences of Moldova, Academy str. 5, MD2028 Chisinau, Republic of Moldova

**Keywords:** crystal structure, zinc, benzene-1,3,5-tri­carb­oxy­lic acid, metal–organic framework

## Abstract

The title three-dimensional metal–organic framework (MOF) compound, {(NH_4_)_2_[Zn_2_(C_9_H_3_O_6_)_2_]·2C_5_H_9_NO}_*n*_, features an anionic framework constructed from Zn^2+^ cations and benzene-1,3,5-tri­carboxyl­ate (BTC) organic anions. Charge balance is achieved by outer sphere ammonium cations formed by degradation of di-*n*-butyl­amine in the solvothermal synthesis of the compound. Binuclear {Zn_2_(COO)_2_} entities act as the framework’s secondary building units. Each Zn^II^ atom has a tetrahedral coordination environment with an O_4_ set of donor atoms. The three-dimensional framework adopts a rutile-type topology and channels are filled in an alternating fashion with ordered and disordered 1-methyl­pyrrolidin-2-one solvent mol­ecules and ammonium cations. The latter are held in the channels *via* four N—H⋯O hydrogen bonds, including three with the benzene-1,3,5-tri­carboxyl­ate ligands of the anionic framework and one with a 1-methyl­pyrrolidin-2-one solvent mol­ecule.

## Abstract 2

The title three-dimensional metal–organic framework (MOF) material features an anionic framework constructed from Zn^2+^ cations and benzene-1,3,5-tri­carboxyl­ate (BTC) organic anions. Charge balance is achieved by outer sphere ammonium cations formed by degradation of di-*n*-butyl­amine in the solvothermal synthesis of the material.

## Chemical context   

1,3,5-Benzene­tri­carb­oxy­lic acid (H_3_BTC) has proved its efficacy as a versatile and powerful ligand for the construction of metal–organic frameworks (MOFs). Its three carboxyl­ate groups and benzene ring can act as short and long bridges between metal ions, leading to three-dimensional assemblies with a large structural diversity (Eddaoudi *et al.*, 2001 ▸; Almeida Paz & Klinowski, 2004 ▸; Liu *et al.*, 2007 ▸). Since 1997 (Yaghi *et al.*, 1997 ▸), the coordination chemistry of zinc ions and BTC ligands has represented one of the most extensively explored systems in efforts to synthesize new porous materials. The various aspects of the Zn–BTC system continue to being investigated, and diverse MOF structures have been reported. The published results reveal that the variation of starting compositions, solvents and templates as well as reaction conditions are significant and can result in the formation of completely different metal–organic framework compounds. A base is needed for deprotonation of H_3_BTC so that it can make use of its full coordination capacity. This base should have a low affinity for binding to metal ions to avoid competition with BTC, especially if the aim is the synthesis of porous materials. A wide range of different solvent systems and reaction conditions have been used in the construction of new coordination networks, including the use of ionothermal techniques (Xu *et al.*, 2007 ▸), and conducting reactions in the presence of different surfactants as reaction media (Gao *et al.*, 2014 ▸).

In our recent work (Ordonez *et al.*, 2014 ▸), we reported 13 different Zn–BTC coordination networks that were formed as a result of the use of different cations as framework templates. Generally, only one type of secondary building unit (SBU) is observed in one compound; however, data from our and other groups (Ordonez *et al.*, 2014 ▸; Xie, 2013 ▸; Hao *et al.*, 2012 ▸) have shown the possibility of different SBUs in a single self-assembled system which can, in turn, result in distinct frameworks and topologies. In some cases, hydro­thermal reaction conditions lead to decomposition of solvents or bases (Burrows *et al.*, 2005 ▸), and fixation of the decomposition products in the systems can result in unexpected guests such as ammonium cations (Ordonez *et al.*, 2014 ▸). Herein we report the structure of a new three-dimensional Zn–BTC MOF obtained serendipitously by reaction of the H_3_BTC ligand with zinc nitrate hexa­hydrate using 1-methyl­pyrrolidin-2-one (NMP) as a solvent and di-(*n*-but­yl)amine as a base and a framework template. The main product of the reaction was the {Zn-BTC}{*n*-Bu_2_NH_2_} MOF, but a few single crystals of title compound were found as a byproduct.
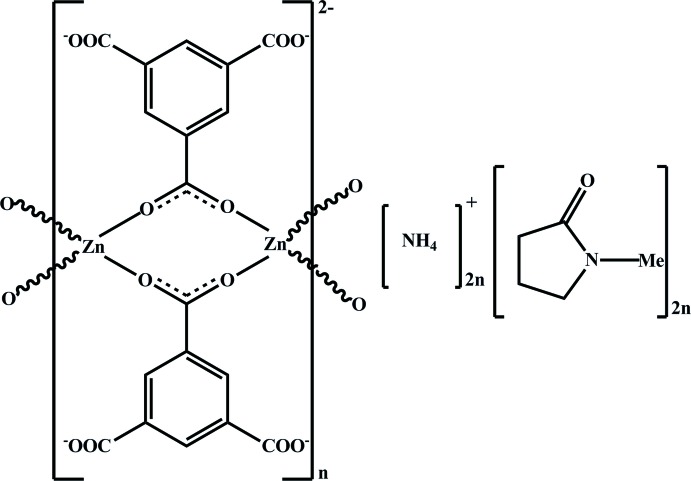


## Structural commentary   

**Figure 1:**
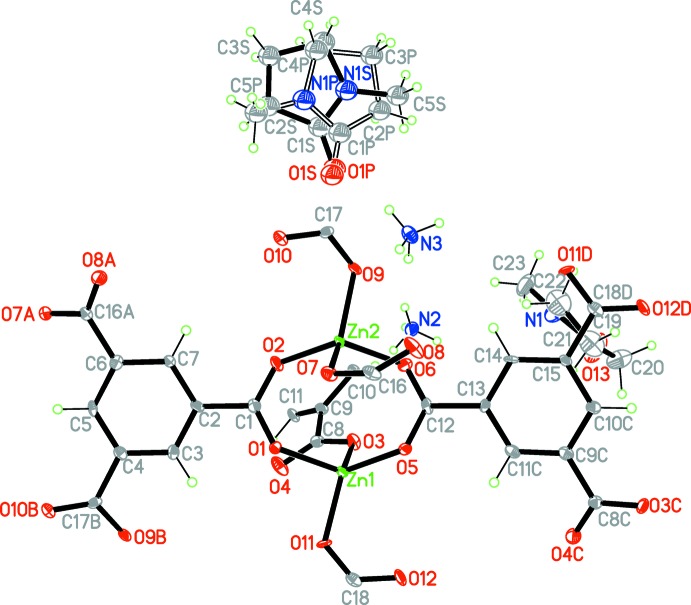
A portion of the crystal structure of the title complex, displaying the atomic labeling. Displacement ellipsoids are drawn at the 50% probability level. [Symmetry codes: (A) 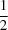 + *x*, 2 − *y*, 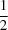 + *z*; (B) 1 + *x*, *y*, *z*; (C) *x* − 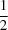, 1 − *y*, *z* − 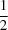; (D) *x* − 1, *y*, *z*.]

**Figure 2:**
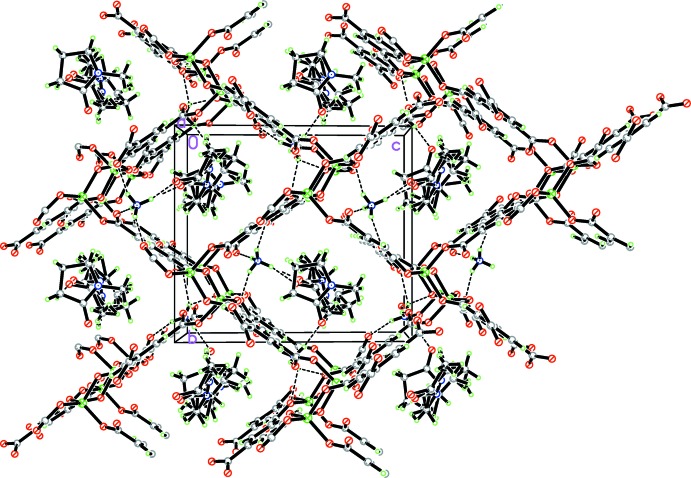
Three-dimensional structure in the unit cell viewed along the *a* axis. Hydrogen-bonding inter­actions are shown as dashed lines. C-bound H atoms in coordination network are omitted for clarity.

The asymmetric unit of the title compound, {(NH_4_)_2_[Zn_2_(C_9_H_3_O_6_)_2_]·2C_5_H_9_NO}_*n*_, contains two Zn^II^ cations, two ammonium cations, two NMP mol­ecules and two BTC residues (Fig. 1 ▸). The compound has a three-dimensional structure constructed from dimeric zinc carboxyl­ate entities and BTC linkers (Fig. 2 ▸). The two zinc ions form a unit with six carboxyl­ate units from the two symmetry-independent BTC ligands, and four additional BTC units created by the glide operations and translations. Each of the Zn^II^ cations exhibits an O_4_ coordination set defined by four oxygen atoms of four coordinating BTC residues. The Zn—O distances range within 1.927 (5)–1.982 (5) Å for Zn1 and 1.926 (5)–1.969 (5) Å for Zn2. Of the six BTC residues around the Zn_2_ units, two act in bidentate bridging modes, and combine the two crystallographically unique Zn^II^ ions in the binuclear cluster {Zn_2_(COO)_2_} that acts as the SBU in this compound. All of the other carb­oxy­lic oxygen atoms coordinate in a monodentate fashion (Fig. 1 ▸). The Zn1⋯Zn2 separation within the SBU is 3.542 (5) Å. The connection of alternating zinc carboxyl­ate units and BTC linkers results in an infinite three-dimensional (3,6)-connected net, which leads to the framework having the same topology as rutile, TiO_2_.

As a result of the lower symmetry of the SBU, the title compound crystallizes in a reduced symmetry space group (*Pn*) compared to rutile (*P*4_2_/*mnm*). Like other Zn–BTC frameworks with rtl-topology (Xie *et al.*, 2005 ▸; Ordonez *et al.*, 2014 ▸), this framework is also porous. There are rectangular channels paralle to the [100] axis, with an approximate dimension of 7.472 x 9.543 Å in which per asymmetric unit two ammonium cations and two NMP mol­ecules (ordered and disordered ones) reside (Fig. 2 ▸). Seven hydrogen-bonding inter­actions are observed between both of the ammonium cations and the carb­oxy­lic framework, N⋯O distances being in the range 2.713 (7)–3.104 (7) Å; two link each of the ammonium cations with each an NMP mol­ecule (Table 1 ▸). The source of the ammonium cations is considered to be from the degradation of di-(*n*-but­yl)amine during the reaction.

## Database survey   

A literature overview (Xu *et al.*, 2007 ▸) reported 41 different Zn–BTC MOFs with a total of 13 types of connectivity modes of BTC with Zn. The 13 modes span all of the possible features of bonds between carb­oxy­lic groups and Zn atoms. Modes with bimetallic Zn coordination were most frequently found, followed by modes with three Zn and with four Zn atoms. A search of the CSD (Groom *et al.*, 2016 ▸; *ConQuest* 1.18, Version 5.37, updates November, 2015) for structures reported after 2007 revealed at least 60 additional {Zn–BTC} carb­oxy­lic networks. The title compound occupies a place in the reticular series of the complexes {Zn–BTC}{Base} for Base = Me_2_NH_2_^+^, Et_2_NH_2_^+^, *n*-Bu_2_NH_2_^+^, Et_3_NH^+^, (PhCH_2_)Me_3_N^+^, and BMIM = 1-butyl-3-methyl­imidazole (Ordonez *et al.*, 2014 ▸). As a result of the size of the templates, the reticular networks differ by the packing modes of the cations in the channels, and correspondingly by channel size within the framework. {Zn/Cd–BTC} networks with the same rtl topology have also been reported (Xie *et al.*, 2005 ▸; Zhao *et al.*, 2007 ▸).

## Synthesis and crystallization   

A mixture of Zn(NO_3_)_2_·6H_2_O (0.343 g, 1.15 mmol), H_3_BTC (0.244g, 1.16 mmol), di-(*n*-but­yl)amine (0.142 g, 1.10 mmol), and 1-methyl­pyrrolidin-2-one (NMP, 10 mL) was prepared in a capped vial. The solution was transferred to a 23 mL Teflon-lined acid digestion vessel and placed in an oven at 423 K for four days. The crystals produced were collected in a vial, washed with fresh NMP, and sonicated to remove impurities from the crystals. The main product of the reaction was the MOF {Zn–BTC}{*n*-Bu_2_NH_2_}; only few single crystals of the title compound were found as a byproduct. Those crystals were plate shaped and colorless. Synthetic details are given in Ordonez *et al.* (2014 ▸).

## Refinement details   

Crystal data, data collection and structure refinement details are summarized in Table 2 ▸. C-bound H atoms were calculated in geometrically idealized positions and refined riding on their parent atoms, with *U*_iso_(H) = 1.2*U*_eq_(C) (aromatic) and 1.5*U*_eq_(C) (meth­yl), and with C—H = 0.95 Å (aromatic) and 0.98 Å (meth­yl). The methyl H atoms were allowed to rotate around the corresponding C—C bond. N-bound H atoms in ammonium cations were found in a difference map and refined using geometrical restraints to fix the N—H distances, and with an isotropic displacement parameter of *U*_iso_(H) = 1.5*U*_eq_(N). One of the NMP mol­ecules is disordered over two positions with partial occupancies 0.903 (8) and 0.097 (8). The geometries of the major and minor NMP moieties were restrained to be similar using a SAME command. The displacement parameters for the disordered NMP mol­ecule were restrained to be similar to each other using a SIMU command with a standard deviation of 0.01 Å^2^.

## Supplementary Material

Crystal structure: contains datablock(s) I. DOI: 10.1107/S2056989016007027/zl2661sup1.cif

Structure factors: contains datablock(s) I. DOI: 10.1107/S2056989016007027/zl2661Isup2.hkl

CCDC reference: 1476509

Additional supporting information:  crystallographic information; 3D view; checkCIF report

## Figures and Tables

**Table 1:** Hydrogen-bond geometry (Å, °)

*D*—H⋯*A*	*D*—H	H⋯*A*	*D*⋯*A*	*D*—H⋯*A*
N3—H8*N*⋯O1*P*	0.89 (3)	1.60 (7)	2.47 (6)	167 (7)
N3—H8*N*⋯O1*S*	0.89 (3)	1.91 (3)	2.779 (9)	166 (6)
N3—H7*N*⋯O12^i^	0.88 (3)	1.97 (4)	2.786 (6)	154 (6)
N3—H6*N*⋯O9	0.87 (3)	2.03 (3)	2.867 (7)	161 (6)
N3—H5*N*⋯O4^ii^	0.86 (3)	1.94 (3)	2.800 (7)	174 (6)
N2—H4*N*⋯O13^iii^	0.86 (3)	1.85 (3)	2.713 (7)	173 (6)
N2—H3*N*⋯O11^i^	0.88 (3)	2.24 (4)	3.025 (7)	148 (6)
N2—H3*N*⋯O1^i^	0.88 (3)	2.41 (5)	3.104 (7)	136 (6)
N2—H2*N*⋯O8^iv^	0.88 (3)	1.91 (4)	2.737 (7)	156 (6)
N2—H1*N*⋯O10^v^	0.88 (3)	1.97 (3)	2.825 (7)	163 (6)

Symmetry codes: (i) 
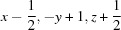
; (ii) 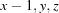; (iii) 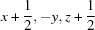; (iv) 
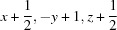
; (v) 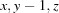.

**Table 2:** Experimental details

Crystal data	
Chemical formula	(NH_4_)_2_[Zn_2_(C_9_H_3_O_6_)_2_]·2C_5_H_9_NO
*M*_r_	779.31
Crystal system, space group	Monoclinic, *P* *n*
Temperature (K)	100
*a*, *b*, *c* (Å)	9.470 (4), 12.351 (5), 13.575 (5)
β (°)	94.327 (5)
*V* (Å^3^)	1583.2 (10)
*Z*	2
Radiation type	Mo *K*α
μ (mm^−1^)	1.59
Crystal size (mm)	0.45 × 0.35 × 0.25

Data collection	
Diffractometer	Bruker *SMART* APEXII CCD area-detector
Absorption correction	Multi-scan (*SADABS*; Bruker, 2009 ▸)
*T*_min_, *T*_max_	0.628, 0.784
No. of measured, independent and observed [*I* > 2σ(*I*)] reflections	13257, 6013, 5263
*R*_int_	0.038
(sin θ/λ)_max_ (Å^−1^)	0.617

Refinement	
*R*[*F* ^2^ > 2σ(*F* ^2^)], *wR*(*F* ^2^), *S*	0.033, 0.068, 0.99
No. of reflections	6013
No. of parameters	525
No. of restraints	236
H-atom treatment	H atoms treated by a mixture of independent and constrained refinement
Δρ_max_, Δρ_min_ (e Å^−3^)	0.38, −0.33
Absolute structure	Refined as an inversion twin.
Absolute structure parameter	0.102 (18)

Computer programs: *APEX2* (Bruker, 2014 ▸), *SAINT-Plus* (Bruker, 2009 ▸), *SHELXTL* (Sheldrick, 2008 ▸), *SHELXL2014* (Sheldrick, 2015 ▸), *ORTEP-3 for Windows* (Farrugia, 2012 ▸) and *publCIF* (Westrip, 2010 ▸).

## supplementary crystallographic information

### Crystal data

**Table:**

(NH_4_)_2_[Zn_2_(C_9_H_3_O_6_)_2_]·2C_5_H_9_NO	*F*(000) = 800
*M**_r_* = 779.31	*D*_x_ = 1.635 Mg m^−^^3^
Monoclinic, *P**n*	Mo *K*α radiation, λ = 0.71073 Å
*a* = 9.470 (4) Å	Cell parameters from 3722 reflections
*b* = 12.351 (5) Å	θ = 4.3–26.2°
*c* = 13.575 (5) Å	µ = 1.59 mm^−^^1^
β = 94.327 (5)°	*T* = 100 K
*V* = 1583.2 (10) Å^3^	Prism, colorless
*Z* = 2	0.45 × 0.35 × 0.25 mm

### Data collection

**Table:**

Bruker SMART APEXII CCD area-detector diffractometer	5263 reflections with *I* > 2σ(*I*)
phi and ω scans	*R*_int_ = 0.038
Absorption correction: multi-scan (*SADABS*; Bruker, 2009)	θ_max_ = 26.0°, θ_min_ = 4.3°
*T*_min_ = 0.628, *T*_max_ = 0.784	*h* = −11→11
13257 measured reflections	*k* = −15→15
6013 independent reflections	*l* = −16→16

### Refinement

**Table:**

Refinement on *F*^2^	Hydrogen site location: mixed
Least-squares matrix: full	H atoms treated by a mixture of independent and constrained refinement
*R*[*F*^2^ > 2σ(*F*^2^)] = 0.033	*w* = 1/[σ^2^(*F*_o_^2^) + (0.029*P*)^2^] where *P* = (*F*_o_^2^ + 2*F*_c_^2^)/3
*wR*(*F*^2^) = 0.068	(Δ/σ)_max_ = 0.002
*S* = 0.99	Δρ_max_ = 0.38 e Å^−^^3^
6013 reflections	Δρ_min_ = −0.33 e Å^−^^3^
525 parameters	Absolute structure: Refined as an inversion twin.
236 restraints	Absolute structure parameter: 0.102 (18)

### Special details

**Table:**

Geometry. All esds (except the esd in the dihedral angle between two l.s. planes) are estimated using the full covariance matrix. The cell esds are taken into account individually in the estimation of esds in distances, angles and torsion angles; correlations between esds in cell parameters are only used when they are defined by crystal symmetry. An approximate (isotropic) treatment of cell esds is used for estimating esds involving l.s. planes.
Refinement. Refined as a 2-component inversion twin.

### Fractional atomic coordinates and isotropic or equivalent isotropic displacement parameters (Å^2^)

**Table:**

	*x*	*y*	*z*	*U*_iso_*/*U*_eq_	Occ. (<1)
Zn1	0.89147 (5)	0.67955 (5)	0.05911 (4)	0.00955 (18)	
Zn2	0.62372 (5)	0.81579 (5)	0.17921 (4)	0.00980 (19)	
O1	0.9514 (6)	0.8118 (3)	0.1336 (4)	0.0115 (12)	
O2	0.8070 (6)	0.8139 (3)	0.2567 (4)	0.0138 (12)	
O3	0.8606 (5)	0.5625 (4)	0.1496 (3)	0.0156 (11)	
O4	1.0719 (5)	0.5942 (4)	0.2254 (4)	0.0213 (11)	
O5	0.7069 (6)	0.6839 (3)	−0.0140 (4)	0.0126 (12)	
O6	0.5633 (6)	0.6808 (3)	0.1113 (4)	0.0129 (12)	
O7	0.6608 (5)	0.9254 (4)	0.0835 (3)	0.0139 (10)	
O8	0.4502 (5)	0.8860 (4)	0.0057 (4)	0.0224 (12)	
O9	0.4623 (5)	0.8233 (3)	0.2583 (4)	0.0127 (12)	
O10	0.5241 (4)	0.9792 (3)	0.3323 (3)	0.0177 (10)	
O11	1.0529 (6)	0.6840 (3)	−0.0191 (4)	0.0134 (12)	
O12	0.9855 (4)	0.5564 (3)	−0.1286 (3)	0.0193 (10)	
O13	0.2466 (6)	0.0795 (4)	0.1181 (3)	0.0482 (14)	
C1	0.9235 (8)	0.8327 (5)	0.2208 (6)	0.0115 (16)	
C2	1.0366 (7)	0.8843 (5)	0.2878 (5)	0.0127 (15)	
C3	1.1771 (7)	0.8723 (5)	0.2683 (5)	0.0110 (15)	
H3	1.2011	0.8307	0.2132	0.013*	
C4	1.2836 (7)	0.9213 (5)	0.3297 (5)	0.0115 (14)	
C5	1.2461 (7)	0.9836 (5)	0.4080 (5)	0.0113 (14)	
H5	1.3177	1.0186	0.4492	0.014*	
C6	1.1059 (7)	0.9959 (5)	0.4271 (5)	0.0134 (15)	
C7	0.9997 (8)	0.9460 (5)	0.3666 (5)	0.0120 (15)	
H7	0.9031	0.9544	0.3795	0.014*	
C8	0.9530 (7)	0.5524 (5)	0.2213 (5)	0.0128 (15)	
C9	0.9120 (7)	0.4837 (5)	0.3048 (5)	0.0134 (15)	
C10	0.7684 (8)	0.4637 (5)	0.3179 (5)	0.0129 (14)	
H10	0.6968	0.4899	0.2710	0.015*	
C11	1.0130 (8)	0.4415 (6)	0.3723 (6)	0.0147 (15)	
H11	1.1102	0.4529	0.3628	0.018*	
C12	0.5904 (8)	0.6637 (5)	0.0231 (6)	0.0113 (16)	
C13	0.4770 (7)	0.6170 (5)	−0.0465 (5)	0.0097 (15)	
C14	0.3341 (7)	0.6340 (5)	−0.0316 (5)	0.0114 (15)	
H14	0.3079	0.6720	0.0251	0.014*	
C15	0.2317 (7)	0.5949 (5)	−0.1000 (5)	0.0108 (14)	
C16	0.5650 (8)	0.9342 (6)	0.0106 (6)	0.0158 (16)	
C17	0.4354 (7)	0.9100 (5)	0.3065 (5)	0.0109 (14)	
C18	1.0761 (7)	0.6109 (5)	−0.0831 (5)	0.0133 (14)	
C19	0.2289 (7)	0.1747 (5)	0.0954 (5)	0.0292 (14)	
C20	0.1892 (9)	0.2178 (6)	−0.0067 (5)	0.0386 (17)	
H20A	0.0926	0.1944	−0.0305	0.046*	
H20B	0.2569	0.1927	−0.0539	0.046*	
C21	0.1961 (8)	0.3402 (6)	0.0056 (6)	0.0429 (18)	
H21A	0.2857	0.3690	−0.0170	0.051*	
H21B	0.1157	0.3754	−0.0328	0.051*	
C22	0.1882 (8)	0.3598 (5)	0.1150 (6)	0.0431 (19)	
H22A	0.2520	0.4195	0.1385	0.052*	
H22B	0.0903	0.3774	0.1306	0.052*	
C23	0.2518 (9)	0.2404 (6)	0.2649 (5)	0.0419 (18)	
H23A	0.1586	0.2397	0.2918	0.063*	
H23B	0.3087	0.2996	0.2952	0.063*	
H23C	0.2997	0.1713	0.2796	0.063*	
N1	0.2347 (6)	0.2561 (4)	0.1594 (4)	0.0314 (13)	
N2	0.6628 (5)	0.0862 (4)	0.4962 (4)	0.0220 (11)	
H1N	0.628 (7)	0.062 (5)	0.438 (3)	0.033*	
H2N	0.749 (4)	0.105 (5)	0.483 (5)	0.033*	
H3N	0.620 (6)	0.145 (4)	0.515 (5)	0.033*	
H4N	0.687 (7)	0.036 (4)	0.539 (4)	0.033*	
N3	0.3320 (5)	0.6331 (4)	0.3316 (4)	0.0197 (11)	
H5N	0.249 (4)	0.624 (5)	0.302 (4)	0.029*	
H6N	0.369 (6)	0.683 (4)	0.296 (4)	0.029*	
H7N	0.393 (6)	0.580 (4)	0.328 (4)	0.029*	
H8N	0.326 (7)	0.660 (5)	0.392 (3)	0.029*	
C1S	0.3028 (9)	0.7644 (7)	0.5883 (6)	0.030 (2)	0.903 (8)
C2S	0.3572 (11)	0.8553 (9)	0.6562 (9)	0.032 (2)	0.903 (8)
H2S1	0.3523	0.9255	0.6209	0.038*	0.903 (8)
H2S2	0.4565	0.8417	0.6812	0.038*	0.903 (8)
C3S	0.2619 (8)	0.8556 (6)	0.7391 (5)	0.0324 (17)	0.903 (8)
H3S1	0.3059	0.8161	0.7970	0.039*	0.903 (8)
H3S2	0.2409	0.9306	0.7592	0.039*	0.903 (8)
C4S	0.1267 (13)	0.7981 (13)	0.6975 (11)	0.036 (2)	0.903 (8)
H4S1	0.0865	0.7520	0.7481	0.043*	0.903 (8)
H4S2	0.0541	0.8506	0.6717	0.043*	0.903 (8)
C5S	0.0917 (12)	0.6515 (11)	0.5659 (10)	0.059 (3)	0.903 (8)
H5S1	0.0244	0.6870	0.5178	0.089*	0.903 (8)
H5S2	0.0396	0.6105	0.6132	0.089*	0.903 (8)
H5S3	0.1522	0.6022	0.5313	0.089*	0.903 (8)
N1S	0.1793 (8)	0.7334 (6)	0.6184 (5)	0.0355 (18)	0.903 (8)
O1S	0.3602 (7)	0.7270 (8)	0.5180 (5)	0.034 (2)	0.903 (8)
C1P	0.258 (6)	0.737 (6)	0.557 (4)	0.034 (4)	0.097 (8)
C2P	0.130 (9)	0.665 (7)	0.572 (8)	0.037 (5)	0.097 (8)
H2P1	0.0741	0.6512	0.5091	0.045*	0.097 (8)
H2P2	0.1604	0.5951	0.6025	0.045*	0.097 (8)
C3P	0.046 (5)	0.730 (5)	0.641 (5)	0.038 (5)	0.097 (8)
H3P1	−0.0295	0.7714	0.6038	0.046*	0.097 (8)
H3P2	0.0018	0.6813	0.6884	0.046*	0.097 (8)
C4P	0.153 (10)	0.806 (13)	0.696 (9)	0.035 (4)	0.097 (8)
H4P1	0.1800	0.7795	0.7637	0.042*	0.097 (8)
H4P2	0.1148	0.8802	0.6996	0.042*	0.097 (8)
C5P	0.392 (9)	0.875 (8)	0.650 (8)	0.029 (9)	0.097 (8)
H5P1	0.4522	0.8697	0.5945	0.043*	0.097 (8)
H5P2	0.4478	0.8561	0.7115	0.043*	0.097 (8)
H5P3	0.3577	0.9499	0.6548	0.043*	0.097 (8)
N1P	0.272 (5)	0.802 (4)	0.635 (4)	0.032 (3)	0.097 (8)
O1P	0.337 (7)	0.732 (8)	0.490 (4)	0.037 (10)	0.097 (8)

### Atomic displacement parameters (Å^2^)

**Table:**

	*U*^11^	*U*^22^	*U*^33^	*U*^12^	*U*^13^	*U*^23^
Zn1	0.0067 (4)	0.0131 (4)	0.0089 (4)	−0.0007 (3)	0.0011 (3)	0.0001 (3)
Zn2	0.0068 (4)	0.0136 (4)	0.0091 (4)	−0.0013 (3)	0.0008 (3)	−0.0002 (3)
O1	0.011 (3)	0.016 (3)	0.008 (3)	−0.0017 (17)	0.000 (2)	−0.0005 (18)
O2	0.007 (3)	0.019 (3)	0.015 (3)	−0.0018 (18)	−0.002 (2)	0.0015 (19)
O3	0.014 (3)	0.020 (2)	0.013 (3)	−0.0026 (19)	−0.002 (2)	0.007 (2)
O4	0.013 (3)	0.026 (3)	0.024 (3)	−0.0063 (19)	−0.001 (2)	0.013 (2)
O5	0.011 (3)	0.019 (3)	0.008 (3)	0.0001 (19)	0.001 (2)	−0.0017 (18)
O6	0.014 (3)	0.014 (3)	0.011 (3)	0.0023 (17)	0.000 (2)	−0.0006 (18)
O7	0.011 (3)	0.018 (2)	0.013 (3)	0.0013 (19)	0.000 (2)	0.0035 (19)
O8	0.011 (3)	0.029 (3)	0.027 (3)	−0.005 (2)	−0.001 (2)	0.013 (2)
O9	0.005 (3)	0.014 (3)	0.019 (3)	0.0011 (17)	0.003 (2)	−0.0007 (19)
O10	0.010 (2)	0.021 (2)	0.022 (2)	−0.0048 (17)	0.0041 (17)	−0.0047 (18)
O11	0.015 (3)	0.017 (3)	0.009 (3)	−0.0019 (18)	0.007 (2)	−0.0046 (18)
O12	0.007 (2)	0.022 (2)	0.029 (3)	−0.0042 (18)	0.0008 (18)	−0.010 (2)
O13	0.083 (4)	0.022 (3)	0.036 (3)	0.008 (2)	−0.015 (3)	0.001 (2)
C1	0.009 (4)	0.008 (3)	0.017 (4)	0.003 (3)	0.000 (3)	0.000 (3)
C2	0.007 (4)	0.014 (3)	0.017 (4)	0.000 (3)	0.003 (3)	−0.001 (3)
C3	0.013 (4)	0.009 (3)	0.011 (4)	−0.002 (3)	−0.002 (3)	0.002 (3)
C4	0.011 (3)	0.011 (3)	0.013 (3)	−0.001 (2)	0.001 (3)	0.003 (3)
C5	0.009 (3)	0.013 (3)	0.011 (3)	−0.003 (2)	−0.005 (2)	0.001 (2)
C6	0.014 (4)	0.012 (3)	0.013 (4)	0.000 (3)	−0.001 (3)	0.000 (3)
C7	0.009 (4)	0.012 (3)	0.015 (4)	−0.001 (3)	0.002 (3)	−0.002 (3)
C8	0.012 (4)	0.015 (3)	0.011 (3)	0.002 (3)	0.001 (3)	0.001 (3)
C9	0.012 (4)	0.014 (3)	0.014 (4)	0.000 (3)	0.003 (3)	−0.001 (3)
C10	0.011 (3)	0.013 (3)	0.016 (3)	−0.004 (2)	0.002 (2)	0.001 (3)
C11	0.009 (4)	0.018 (3)	0.017 (4)	−0.003 (3)	0.001 (3)	0.001 (3)
C12	0.010 (4)	0.011 (3)	0.012 (4)	0.000 (3)	−0.006 (3)	0.004 (3)
C13	0.011 (4)	0.011 (3)	0.007 (4)	−0.001 (3)	−0.002 (3)	−0.001 (3)
C14	0.009 (4)	0.014 (3)	0.013 (4)	0.003 (3)	0.007 (3)	−0.001 (3)
C15	0.007 (3)	0.010 (3)	0.015 (3)	0.001 (2)	0.002 (3)	−0.002 (3)
C16	0.012 (4)	0.016 (3)	0.020 (4)	0.003 (3)	0.005 (3)	0.004 (3)
C17	0.011 (3)	0.016 (3)	0.006 (3)	0.004 (3)	0.000 (2)	−0.001 (2)
C18	0.011 (3)	0.013 (3)	0.016 (3)	0.000 (2)	0.002 (2)	0.006 (3)
C19	0.039 (4)	0.026 (3)	0.022 (3)	0.009 (3)	0.002 (3)	−0.003 (3)
C20	0.050 (5)	0.040 (4)	0.028 (4)	0.013 (4)	0.010 (3)	0.002 (3)
C21	0.036 (4)	0.044 (5)	0.047 (5)	0.001 (3)	−0.002 (3)	0.020 (4)
C22	0.043 (5)	0.017 (3)	0.069 (5)	0.005 (3)	0.001 (4)	−0.002 (4)
C23	0.053 (5)	0.047 (5)	0.025 (4)	−0.009 (4)	0.001 (3)	−0.014 (3)
N1	0.045 (4)	0.021 (3)	0.029 (3)	−0.001 (2)	0.008 (3)	−0.003 (2)
N2	0.018 (3)	0.019 (3)	0.028 (3)	0.005 (2)	−0.001 (2)	−0.011 (2)
N3	0.014 (3)	0.018 (3)	0.027 (3)	0.001 (2)	−0.001 (2)	0.003 (2)
C1S	0.026 (4)	0.034 (4)	0.029 (4)	0.005 (3)	0.002 (3)	0.007 (4)
C2S	0.032 (5)	0.032 (5)	0.030 (4)	0.002 (4)	−0.001 (4)	−0.002 (4)
C3S	0.034 (4)	0.034 (4)	0.029 (4)	0.004 (3)	0.003 (3)	−0.002 (3)
C4S	0.032 (5)	0.040 (4)	0.034 (4)	0.007 (4)	0.007 (4)	0.002 (4)
C5S	0.053 (7)	0.063 (7)	0.059 (6)	−0.016 (6)	−0.012 (6)	−0.015 (5)
N1S	0.028 (4)	0.041 (4)	0.039 (4)	−0.001 (3)	0.005 (3)	0.000 (3)
O1S	0.032 (4)	0.041 (4)	0.030 (4)	0.010 (3)	−0.005 (3)	−0.008 (4)
C1P	0.028 (8)	0.038 (8)	0.035 (8)	0.004 (8)	0.000 (8)	0.004 (8)
C2P	0.031 (9)	0.042 (9)	0.038 (9)	−0.002 (9)	0.001 (9)	0.000 (9)
C3P	0.033 (8)	0.042 (8)	0.039 (8)	−0.002 (8)	0.005 (8)	0.002 (8)
C4P	0.031 (7)	0.039 (7)	0.034 (7)	0.003 (7)	0.005 (7)	0.001 (7)
C5P	0.031 (15)	0.033 (15)	0.022 (15)	0.005 (15)	−0.004 (15)	−0.003 (14)
N1P	0.030 (6)	0.035 (6)	0.032 (6)	0.004 (6)	0.001 (6)	0.001 (6)
O1P	0.041 (17)	0.038 (16)	0.032 (17)	0.017 (16)	−0.006 (16)	−0.001 (17)

### Geometric parameters (Å, º)

**Table:**

Zn1—O3	1.933 (5)	C20—H20A	0.9900
Zn1—O11	1.927 (5)	C20—H20B	0.9900
Zn1—O5	1.944 (5)	C21—C22	1.513 (11)
Zn1—O1	1.982 (5)	C21—H21A	0.9900
Zn2—O7	1.926 (5)	C21—H21B	0.9900
Zn2—O9	1.935 (5)	C22—N1	1.469 (8)
Zn2—O2	1.960 (5)	C22—H22A	0.9900
Zn2—O6	1.969 (5)	C22—H22B	0.9900
O1—C1	1.259 (9)	C23—N1	1.443 (9)
O2—C1	1.261 (9)	C23—H23A	0.9800
O3—C8	1.265 (8)	C23—H23B	0.9800
O4—C8	1.237 (8)	C23—H23C	0.9800
O5—C12	1.272 (9)	N2—H1N	0.88 (3)
O6—C12	1.261 (9)	N2—H2N	0.88 (3)
O7—C16	1.296 (8)	N2—H3N	0.88 (3)
O8—C16	1.237 (8)	N2—H4N	0.86 (3)
O9—C17	1.290 (7)	N3—H5N	0.86 (3)
O10—C17	1.231 (7)	N3—H6N	0.87 (3)
O11—C18	1.283 (8)	N3—H7N	0.88 (3)
O12—C18	1.222 (8)	N3—H8N	0.89 (3)
O13—C19	1.224 (7)	C1S—O1S	1.223 (11)
C1—C2	1.494 (10)	C1S—N1S	1.325 (8)
C2—C7	1.381 (9)	C1S—C2S	1.517 (14)
C2—C3	1.384 (9)	C2S—C3S	1.495 (13)
C3—C4	1.397 (9)	C2S—H2S1	0.9900
C3—H3	0.9500	C2S—H2S2	0.9900
C4—C5	1.380 (9)	C3S—C4S	1.533 (13)
C4—C17^i^	1.501 (9)	C3S—H3S1	0.9900
C5—C6	1.381 (9)	C3S—H3S2	0.9900
C5—H5	0.9500	C4S—N1S	1.457 (11)
C6—C7	1.392 (10)	C4S—H4S1	0.9900
C6—C16^ii^	1.500 (10)	C4S—H4S2	0.9900
C7—H7	0.9500	C5S—N1S	1.459 (11)
C8—C9	1.490 (9)	C5S—H5S1	0.9800
C9—C11	1.376 (10)	C5S—H5S2	0.9800
C9—C10	1.407 (9)	C5S—H5S3	0.9800
C10—C15^iii^	1.395 (9)	C1P—O1P	1.22 (3)
C10—H10	0.9500	C1P—N1P	1.33 (3)
C11—C13^iii^	1.382 (9)	C1P—C2P	1.52 (3)
C11—H11	0.9500	C2P—C3P	1.50 (3)
C12—C13	1.492 (10)	C2P—H2P1	0.9900
C13—C11^iv^	1.382 (9)	C2P—H2P2	0.9900
C13—C14	1.400 (9)	C3P—C4P	1.53 (3)
C14—C15	1.378 (9)	C3P—H3P1	0.9900
C14—H14	0.9500	C3P—H3P2	0.9900
C15—C10^iv^	1.395 (9)	C4P—N1P	1.46 (3)
C15—C18^v^	1.521 (9)	C4P—H4P1	0.9900
C16—C6^vi^	1.500 (10)	C4P—H4P2	0.9900
C17—C4^v^	1.501 (9)	C5P—N1P	1.46 (3)
C18—C15^i^	1.521 (9)	C5P—H5P1	0.9800
C19—N1	1.327 (7)	C5P—H5P2	0.9800
C19—C20	1.505 (9)	C5P—H5P3	0.9800
C20—C21	1.522 (9)		
			
O3—Zn1—O11	122.4 (2)	H21A—C21—H21B	108.8
O3—Zn1—O5	99.9 (2)	N1—C22—C21	103.1 (5)
O11—Zn1—O5	116.0 (2)	N1—C22—H22A	111.1
O3—Zn1—O1	110.1 (2)	C21—C22—H22A	111.1
O11—Zn1—O1	92.8 (2)	N1—C22—H22B	111.1
O5—Zn1—O1	116.7 (2)	C21—C22—H22B	111.1
O7—Zn2—O9	122.16 (19)	H22A—C22—H22B	109.1
O7—Zn2—O2	99.8 (2)	N1—C23—H23A	109.5
O9—Zn2—O2	114.1 (2)	N1—C23—H23B	109.5
O7—Zn2—O6	109.9 (2)	H23A—C23—H23B	109.5
O9—Zn2—O6	94.9 (2)	N1—C23—H23C	109.5
O2—Zn2—O6	117.1 (2)	H23A—C23—H23C	109.5
C1—O1—Zn1	125.2 (5)	H23B—C23—H23C	109.5
C1—O2—Zn2	123.8 (5)	C19—N1—C23	123.0 (6)
C8—O3—Zn1	115.7 (4)	C19—N1—C22	113.3 (5)
C12—O5—Zn1	124.5 (5)	C23—N1—C22	122.1 (6)
C12—O6—Zn2	120.9 (5)	H1N—N2—H2N	101 (6)
C16—O7—Zn2	114.9 (4)	H1N—N2—H3N	113 (6)
C17—O9—Zn2	121.0 (4)	H2N—N2—H3N	107 (6)
C18—O11—Zn1	122.4 (4)	H1N—N2—H4N	115 (6)
O1—C1—O2	125.3 (7)	H2N—N2—H4N	97 (6)
O1—C1—C2	117.9 (7)	H3N—N2—H4N	120 (6)
O2—C1—C2	116.8 (7)	H5N—N3—H6N	103 (6)
C7—C2—C3	120.7 (6)	H5N—N3—H7N	117 (6)
C7—C2—C1	119.7 (7)	H6N—N3—H7N	102 (6)
C3—C2—C1	119.6 (6)	H5N—N3—H8N	111 (6)
C2—C3—C4	120.1 (6)	H6N—N3—H8N	107 (6)
C2—C3—H3	120.0	H7N—N3—H8N	115 (6)
C4—C3—H3	120.0	O1S—C1S—N1S	125.9 (9)
C5—C4—C3	119.0 (7)	O1S—C1S—C2S	127.0 (9)
C5—C4—C17^i^	121.3 (6)	N1S—C1S—C2S	107.1 (7)
C3—C4—C17^i^	119.6 (6)	C1S—C2S—C3S	105.2 (7)
C4—C5—C6	120.9 (6)	C1S—C2S—H2S1	110.7
C4—C5—H5	119.6	C3S—C2S—H2S1	110.7
C6—C5—H5	119.6	C1S—C2S—H2S2	110.7
C7—C6—C5	120.2 (6)	C3S—C2S—H2S2	110.7
C7—C6—C16^ii^	119.0 (6)	H2S1—C2S—H2S2	108.8
C5—C6—C16^ii^	120.8 (6)	C4S—C3S—C2S	104.9 (7)
C2—C7—C6	119.2 (7)	C4S—C3S—H3S1	110.8
C2—C7—H7	120.4	C2S—C3S—H3S1	110.8
C6—C7—H7	120.4	C4S—C3S—H3S2	110.8
O4—C8—O3	124.6 (6)	C2S—C3S—H3S2	110.8
O4—C8—C9	119.5 (6)	H3S1—C3S—H3S2	108.8
O3—C8—C9	115.9 (6)	N1S—C4S—C3S	101.7 (7)
C11—C9—C10	118.6 (7)	N1S—C4S—H4S1	111.4
C11—C9—C8	120.9 (7)	C3S—C4S—H4S1	111.4
C10—C9—C8	120.4 (6)	N1S—C4S—H4S2	111.4
C15^iii^—C10—C9	119.6 (6)	C3S—C4S—H4S2	111.4
C15^iii^—C10—H10	120.2	H4S1—C4S—H4S2	109.3
C9—C10—H10	120.2	N1S—C5S—H5S1	109.5
C13^iii^—C11—C9	121.9 (7)	N1S—C5S—H5S2	109.5
C13^iii^—C11—H11	119.0	H5S1—C5S—H5S2	109.5
C9—C11—H11	119.0	N1S—C5S—H5S3	109.5
O5—C12—O6	126.0 (7)	H5S1—C5S—H5S3	109.5
O5—C12—C13	115.4 (7)	H5S2—C5S—H5S3	109.5
O6—C12—C13	118.5 (7)	C1S—N1S—C5S	122.2 (8)
C11^iv^—C13—C14	119.6 (6)	C1S—N1S—C4S	115.4 (8)
C11^iv^—C13—C12	119.9 (7)	C5S—N1S—C4S	121.8 (8)
C14—C13—C12	120.5 (6)	O1P—C1P—N1P	126 (4)
C15—C14—C13	119.3 (6)	O1P—C1P—C2P	128 (4)
C15—C14—H14	120.4	N1P—C1P—C2P	106 (3)
C13—C14—H14	120.4	C3P—C2P—C1P	104 (3)
C14—C15—C10^iv^	121.0 (6)	C3P—C2P—H2P1	110.9
C14—C15—C18^v^	119.6 (6)	C1P—C2P—H2P1	110.9
C10^iv^—C15—C18^v^	119.4 (6)	C3P—C2P—H2P2	111.0
O8—C16—O7	124.3 (7)	C1P—C2P—H2P2	111.0
O8—C16—C6^vi^	120.8 (7)	H2P1—C2P—H2P2	109.0
O7—C16—C6^vi^	114.9 (6)	C4P—C3P—C2P	105 (3)
O10—C17—O9	124.3 (6)	C4P—C3P—H3P1	110.7
O10—C17—C4^v^	121.3 (6)	C2P—C3P—H3P1	110.7
O9—C17—C4^v^	114.4 (6)	C4P—C3P—H3P2	110.7
O12—C18—O11	125.5 (6)	C2P—C3P—H3P2	110.7
O12—C18—C15^i^	120.2 (6)	H3P1—C3P—H3P2	108.8
O11—C18—C15^i^	114.2 (6)	C3P—C4P—N1P	102 (3)
O13—C19—N1	124.4 (6)	C3P—C4P—H4P1	111.3
O13—C19—C20	126.3 (6)	N1P—C4P—H4P1	111.3
N1—C19—C20	109.3 (6)	C3P—C4P—H4P2	111.3
C19—C20—C21	104.2 (6)	N1P—C4P—H4P2	111.3
C19—C20—H20A	110.9	H4P1—C4P—H4P2	109.2
C21—C20—H20A	110.9	N1P—C5P—H5P1	109.5
C19—C20—H20B	110.9	N1P—C5P—H5P2	109.5
C21—C20—H20B	110.9	H5P1—C5P—H5P2	109.5
H20A—C20—H20B	108.9	N1P—C5P—H5P3	109.5
C22—C21—C20	105.2 (6)	H5P1—C5P—H5P3	109.5
C22—C21—H21A	110.7	H5P2—C5P—H5P3	109.5
C20—C21—H21A	110.7	C1P—N1P—C5P	122 (4)
C22—C21—H21B	110.7	C1P—N1P—C4P	116 (3)
C20—C21—H21B	110.7	C5P—N1P—C4P	122 (4)

Symmetry codes: (i) *x*+1, *y*, *z*; (ii) *x*+1/2, −*y*+2, *z*+1/2; (iii) *x*+1/2, −*y*+1, *z*+1/2; (iv) *x*−1/2, −*y*+1, *z*−1/2; (v) *x*−1, *y*, *z*; (vi) *x*−1/2, −*y*+2, *z*−1/2.

### Hydrogen-bond geometry (Å, º)

**Table:**

*D*—H···*A*	*D*—H	H···*A*	*D*···*A*	*D*—H···*A*
N3—H8*N*···O1*P*	0.89 (3)	1.60 (7)	2.47 (6)	167 (7)
N3—H8*N*···O1*S*	0.89 (3)	1.91 (3)	2.779 (9)	166 (6)
N3—H7*N*···O12^vii^	0.88 (3)	1.97 (4)	2.786 (6)	154 (6)
N3—H6*N*···O9	0.87 (3)	2.03 (3)	2.867 (7)	161 (6)
N3—H5*N*···O4^v^	0.86 (3)	1.94 (3)	2.800 (7)	174 (6)
N2—H4*N*···O13^viii^	0.86 (3)	1.85 (3)	2.713 (7)	173 (6)
N2—H3*N*···O11^vii^	0.88 (3)	2.24 (4)	3.025 (7)	148 (6)
N2—H3*N*···O1^vii^	0.88 (3)	2.41 (5)	3.104 (7)	136 (6)
N2—H2*N*···O8^iii^	0.88 (3)	1.91 (4)	2.737 (7)	156 (6)
N2—H1*N*···O10^ix^	0.88 (3)	1.97 (3)	2.825 (7)	163 (6)

Symmetry codes: (iii) *x*+1/2, −*y*+1, *z*+1/2; (v) *x*−1, *y*, *z*; (vii) *x*−1/2, −*y*+1, *z*+1/2; (viii) *x*+1/2, −*y*, *z*+1/2; (ix) *x*, *y*−1, *z*.
